# An unexpected finding of large bowel obstruction from colonic diaphragm disease following prolonged use of indomethacin

**DOI:** 10.1093/jscr/rjaf1074

**Published:** 2026-01-19

**Authors:** Isabella Zappala, Joseph Do Woong Choi, Evangeline Woodford, Raymond Kwok, Peter Dutton

**Affiliations:** Department of Surgery, Westmead Hospital, Corner of Hawkesbury Road and Darcy Roads, Westmead New South Wales, 2145, Australia; Department of Surgery, Westmead Hospital, Corner of Hawkesbury Road and Darcy Roads, Westmead New South Wales, 2145, Australia; Department of Surgery, Norwest Private Hospital, 11 Norbrik Drive, Bella Vista, New South Wales, 2153, Australia; Faculty of Medicine and Health, The University of Sydney, Science Rd, Camperdown, New South Wales, 2050, Australia; Department of Surgery, Westmead Hospital, Corner of Hawkesbury Road and Darcy Roads, Westmead New South Wales, 2145, Australia; Department of Surgery, Norwest Private Hospital, 11 Norbrik Drive, Bella Vista, New South Wales, 2153, Australia; Department of Gastroenterology, Norwest Private Hospital, 11 Norbrik Drive, Bella Vista, New South Wales, 2153, Australia; Department of Surgery, Norwest Private Hospital, 11 Norbrik Drive, Bella Vista, New South Wales, 2153, Australia

**Keywords:** colonic diaphragm disease, NSAID use, hemicolectomy, endoscopic balloon dilatation, Crohn’s disease, gastroenterology, colorectal surgery, surgery

## Abstract

Colonic stricture leading to large bowel obstruction is an uncommon presentation to the emergency department. Strictures are commonly established secondary to diverticular disease, inflammatory bowel disease, malignancy or ischaemic/infective colitis. The formation of a colonic stricture secondary to the chronic use of non-steroidal anti-inflammatory drugs (NSAIDs) is an unusual finding. We present a case of a 48-year-old lady who presented with a large bowel obstruction secondary to NSAID-induced right colonic stricture in a patient who was initially thought to have an index presentation of colonic Crohn’s disease.

## Introduction

Colonic strictures are product of recurrent deposition of fibrous tissue in chronic inflammatory processes described in inflammatory bowel disease (IBD), diverticulitis, ischaemic/infective colitis and colonic malignancy [1]. Similarly, upper gastrointestinal (UGI) strictures develop from pro-inflammatory states, which can be triggered by long-term non-steroidal anti-inflammatory drug (NSAID) use [2]. While NSAIDs are commonly prescribed as first-line analgesics, there is a risk of adverse gastrointestinal (GI) mucosal events when chronically administered [3].

Within the stomach, NSAIDs indirectly cause mucosal injury by inhibiting cyclo-oxygenase-1, which reduces prostaglandin production. Prostaglandins play a crucial role in protecting mucosal cells and regulating acid secretion into the UGI tract, however when this process is inhibited, the integrity of the mucosa is compromised [4]. As a result, GI ulceration occurs, which expeditiously activates an inflammatory response, increasing the risk of stricture formation. Interestingly, the pathophysiology of NSAID-induced lower GI strictures is less established and has been described as a three-hit hypothesis. Firstly, NSAIDs breakdown phospholipid bilayers on the GI mucosal surface, directly damaging mucosal cell mitochondria. Secondly, this decreases ATP intracellularly and promotes calcium efflux and formation of free radicals. Thirdly, there is disruption of normal cellular architecture, and increase in mucosal cell permeability of bile acid, proteolytic enzymes, intestinal bacteria, and toxins leading to cellular death [5]. The colon responds by initiating an inflammatory response that contributes to ulceration and stricture formation.

Proton-pump inhibitors (PPIs), which act to suppress acid secretion assist to reduce the incidence of gastro-duodenal ulcers while on NSAID therapy [6]. PPIs compensate for the loss of prostaglandin mucosal protection, however, are ineffective below the Ligament of Treitz, creating a pro-inflammatory state distal to this site [5].

There are distinguishable endoscopic features of NSAID-derived strictures [7]. If present, they are considered pathognomonic of the condition classified as diaphragm disease [7]. Most cases are reported in the small bowel [8]. Colonic diaphragm disease (CDD) is exceptionally rare with a limited number of cases described since first reported in 1989 [9]. The authors present a unique case of large bowel obstruction from CDD in a patient who was suspected to have newly diagnosed Crohn’s disease.

## Case history

**Figure 1:**
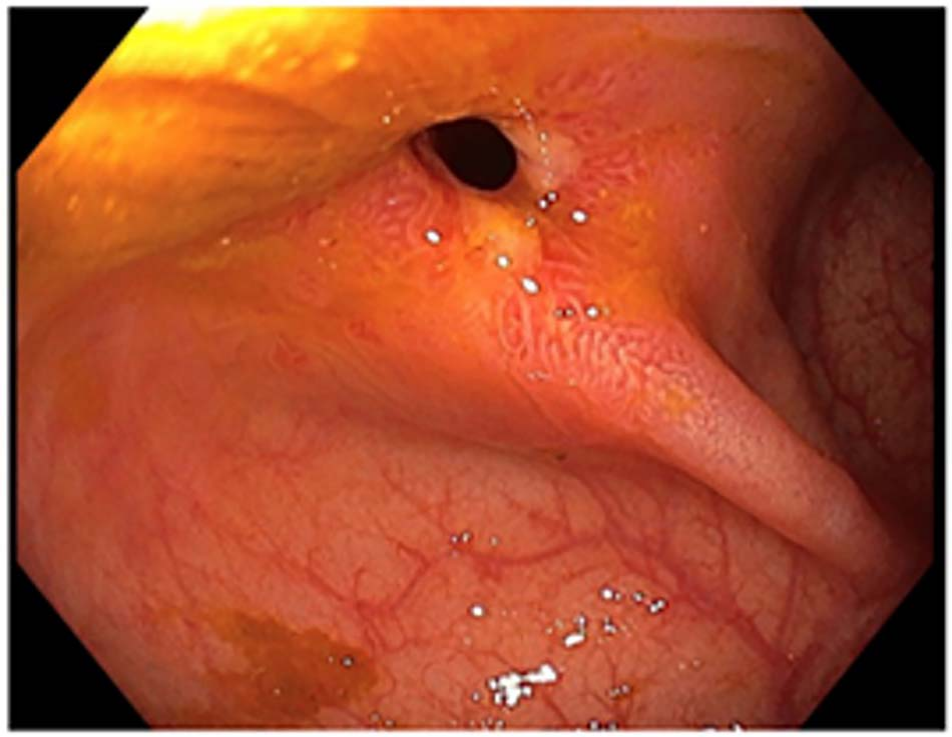
Colonoscopic picture of the diaphragm-like stricture in the ascending colon, 60 cm from the anal verge.

**Figure 2:**
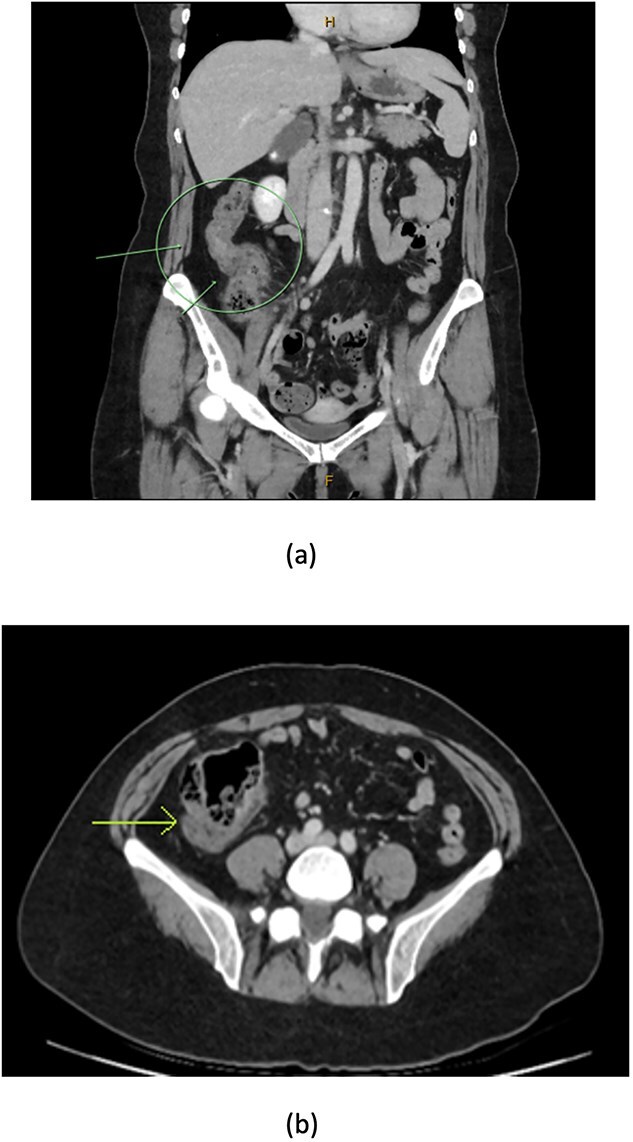
CT coronal (a) and axial (b) views showing thickening of the ascending colon (arrows) with faecalised and distended distal ileum.

**Figure 3:**
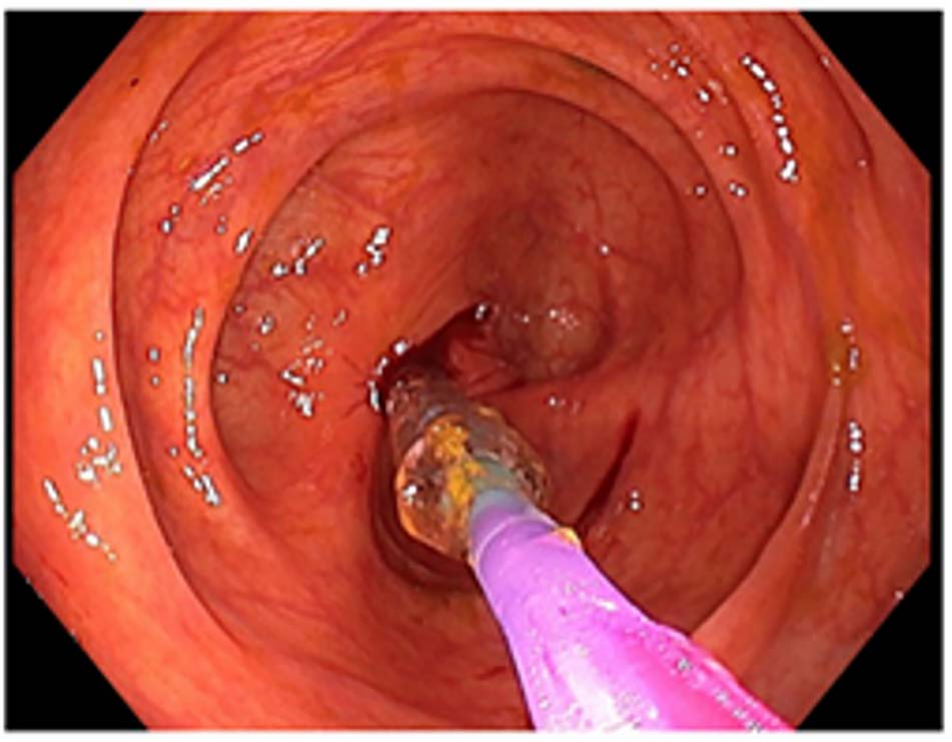
Attempted dilatation with a 6 mm balloon. The procedure was aborted after bleeding commenced.

A 48-year-old lady with a background history of chronic migraines and family history of Crohn’s Disease presented to the emergency department with a three-day history of worsening abdominal pain and obstipation. She underwent a colonoscopy three days prior, which demonstrated shallow ulceration in the rectum and distal colon as well as a web-like stenosis in the ascending colon, 60 cm from the anal verge. The stenotic region was <5 mm in size and was unable to be traversed by the colonoscope, suspicious for a newly diagnosed stricturing Crohn’s disease (Fig. 1). She admitted to taking indomethacin suppositories 100 mg daily over the last two years for her migraines, as well as propranolol, triptan, ondansetron and sertraline. On examination her abdomen was soft, and tender in the right paraumbilical region without peritonism. Her haemoglobin was 94 g/L (115–165 g/L), C reactive protein (CRP) demonstrated a moderate inflammatory process at 40 mg/L (< 5 mg/L). Serum biochemistry and white cells were within normal limits. Computed tomography (CT) demonstrated a stricture in the ascending colon with an evolving proximal large bowel obstruction (Fig. 2). There was faecalisation in the terminal ileum suggestive of an acute on chronic obstruction. There was no suggestion of gastrointestinal perforation, or metastatic disease. The patient was made nil by mouth and prescribed intravenous hydrocortisone 100 milligrams four times a day by the gastroenterologist for suspected Crohn’s disease. However, the patient failed to progress after four days of hydrocortisone. A colonoscopic balloon dilatation was attempted with a 6 mm balloon, however the stricture appeared well-established, and the site began to bleed after the first attempt (Fig. 3). Subsequent attempts were abandoned due to the risk of bowel perforation. The distal end of the stricture was tattooed using spot ink. A colorectal surgeon became involved in her care who recommended a laparoscopic right hemicolectomy.

Intraoperatively, the stricture was identified to be just proximal to the hepatic flexure. There was no macroscopic evidence of enlarged mesocolic lymph nodes or metastatic disease. The right colon was medialised from the caecum to the proximal transverse colon by incising the white line of Toldt and mobilising the hepatic flexure. The ileocolic pedicle was skeletonised and a high transection was performed with an Echelon 60 mm vascular stapler after isolating the duodenum. A mini midline laparotomy was performed to allow for an extra-corporal transection of the terminal ileum, proximal transverse colon and the colonic mesentery after good marginal artery flow was observed via the right branch of the middle colic artery. A side-to-side functional end to end anti-peristaltic ileocolic anastomosis was performed using an Ethicon NTLC 75 stapler in a standard fashion. The patient made an uncomplicated recovery and was discharged after 10 days. Histopathology demonstrated an NSAID-induced web stenosis. There was no evidence of Crohn’s disease in the endoscopic or operative specimens. Resected lymph nodes in the right hemicolectomy specimen showed no pathology or evidence of malignancy. The patient returned for follow-up after four weeks with resolution of abdominal symptoms, and tolerating normal diet.

## Discussion

An NSAID-induced colonic stricture is an unusual and rare phenomenon [10]. A total of eight cases have been documented since the year 2000, with twenty-four documented prior to this period [11]. In this case, the patient presented with abdominal pain and obstipation, concerning for large bowel obstruction. These symptoms were also reported in cases pre-2000. The patient’s symptoms did not resolve following hydrocortisone, and she proceeded to a right hemicolectomy for definitive treatment. The presentation highlights the extent of severity of CDD, a condition that should be considered when investigating for large bowel obstruction.

The extent of diaphragm-like strictures appears to be determined by the duration of NSAID use and class of NSAID. A case–control study performed in 2016 demonstrated that continuous chronic NSAID exposure between 4–12 months was strongly associated with colonic mucosal inflammation [12]. Similar conclusions were made in the small bowel with the severity of mucosal breakdown and injury increasing with the duration of NSAID use. Furthermore, concomitant use of NSAIDs and PPIs at 4–12 months markedly increased the risk of colonic mucosal inflammation compared to patients taking NSAIDs alone. Prolonged use of oral or suppository non-selective classes of NSAIDS contributed most to the formation of colonic strictures [11]. The non-selective class of NSAIDs such as diclofenac, indomethacin and aspirin are proven to be more destructive within the GI tract as they are directly involved in the inhibition of the acid-neutralising prostaglandin [13]. All NSAIDs in these cases were modified release and were more closely associated with stricture formation compared to immediate release NSAIDs.

Crohn’s disease is a major differential to exclude in CDD diagnoses, particularly in this case where there is a family history of Crohn’s disease and endoscopic findings of multiple colonic and rectal ulcers, which are identified in both diseases [8]. Endoscopically, diaphragm-like strictures caused by NSAID-induced inflammation resemble multiple thin, circumferential concentric strictures, with surrounding erosion and sharply demarcated ulcers limited to the mucosa [9]. These findings differ from strictures in Crohn’s disease, which are characterised as luminal narrowings surrounded by longitudinal ulcers of varying mural wall depth, cobblestone appearance of the mucosa and extensive irregular aphthae [14]. Histological characteristics of granulomata, transmural inflammation and fistulating ulcerations favour a diagnosis of Crohn’s disease [15]. Absence of these features favour CDD. Crohn’s disease responds effectively to immune-modulating therapies such as corticosteroids [14]. There is almost no evidence in the literature of the efficacy of corticosteroids in the treatment of CDD [15]. Our patient was initially treated with hydrocortisone 100 mg intravenously four times per day with no improvement of her symptoms or signs. Failure to progress with steroid therapy also favoured CDD diagnosis.

Patient education regarding the effects of NSAIDs on the GI tract is essential in the prevention of NSAID-induced GI stricturing [11]. There is limited data reporting the recurrence rates of CDD in patients no longer using NSAIDs and who have undergone either endoscopic or surgical intervention. Prospective studies following these patients could expand our knowledge of CDD; its long-term effects and recurrence rates without ongoing NSAID exposure.

## References

[1] Rubin DC, Shaker A, Levin MS. Chronic intestinal inflammation: inflammatory bowel disease and colitis-associated colon cancer. Front Immunol 2012;3:107. 22586430 10.3389/fimmu.2012.00107 PMC3347037

[2] Sample JW, Solanki MH, Solanki MH, et al. Nonsteroidal anti-inflammatory drug-induced small bowel strictures (diaphragm disease) - an under-recognized cause of small bowel obstruction. J Gastrointest Surg 2024;28:1430–5. 38871074 10.1016/j.gassur.2024.06.004

[3] Agrawal N . Risk factors for gastrointestinal ulcers caused by nonsteroidal anti-inflammatory drugs (NSAIDs). J Fam Pract 1991;32:619–24. 2040888

[4] Ghlichloo I, Gerriets V. Nonsteroidal Anti-Inflammatory Drugs (NSAIDs). StatPearls. Treasure Island (FL) ineligible companies. 2025. 31613522

[5] Lim YJ, Yang CH. Non-steroidal anti-inflammatory drug-induced enteropathy. Clin Endosc 2012;45:138–44. 22866254 10.5946/ce.2012.45.2.138 PMC3401617

[6] Yang M, He M, Zhao M, et al. Proton pump inhibitors for preventing non-steroidal anti-inflammatory drug induced gastrointestinal toxicity: a systematic review. Curr Med Res Opin 2017;33:973–80. 28076696 10.1080/03007995.2017.1281110

[7] McNally M, Cretu I. A curious case of intestinal diaphragm disease unmasked by perforation of a duodenal ulcer. Case Rep Med 2017;2017:5048345. 28523070 10.1155/2017/5048345 PMC5385904

[8] Wang YZ, Sun G, Cai FC, et al. Clinical features, diagnosis, and treatment strategies of gastrointestinal diaphragm disease associated with nonsteroidal anti-inflammatory drugs. Gastroenterol Res Pract 2016;2016:3679741. 27118967 10.1155/2016/3679741 PMC4826940

[9] Farricielli L, Sanderson DJ. Colonic diaphragm disease: an important NSAID complication to know. Fed Pract 2017;34:38–40. PMC6370415 30766265

[10] Saleem N, Marella HK, Ali B, et al. Colonic diaphragm disease secondary to nonsteroidal anti-inflammatory drug use. Proc (Bayl Univ Med Cent) 2020;33:391–2. 32675960 10.1080/08998280.2020.1732162 PMC7340423

[11] Smith JA, Pineau BC. Endoscopic therapy of NSAID-induced colonic diaphragm disease: two cases and a review of published reports. Gastrointest Endosc 2000;52:120–5. 10882981 10.1067/mge.2000.105979

[12] Tai FWD, McAlindon ME. Non-steroidal anti-inflammatory drugs and the gastrointestinal tract. Clin Med (Lond) 2021;21:131–4. 33762373 10.7861/clinmed.2021-0039 PMC8002800

[13] Qureshi O, Dua A. COX Inhibitors. StatPearls. Treasure Island (FL). 2025. 31747202

[14] Nakase H, Uchino M, Shinzaki S, et al. Evidence-based clinical practice guidelines for inflammatory bowel disease 2020. J Gastroenterol 2021;56:489–526. 33885977 10.1007/s00535-021-01784-1 PMC8137635

[15] Ballal RR, Ahmed T, Ail DA, et al. A rare presentation of idiopathic small bowel diaphragm disease - a case report. Int J Surg Case Rep 2023;112:108966. 37883871 10.1016/j.ijscr.2023.108966 PMC10667883

